# Auditory Long-Range Parvalbumin Cortico-Striatal Neurons

**DOI:** 10.3389/fncir.2020.00045

**Published:** 2020-07-23

**Authors:** Alice Bertero, Hector Zurita, Marc Normandin, Alfonso Junior Apicella

**Affiliations:** Department of Biology, Neurosciences Institute, The University of Texas at San Antonio, San Antonio, TX, United States

**Keywords:** long-range, gabaergic neurons, interneurons, parvalbumin-expressing, auditory cortex, excitation, inhibition, connectivity patterns

## Abstract

Previous studies have shown that cortico-striatal pathways link auditory signals to action-selection and reward-learning behavior through excitatory projections. Only recently it has been demonstrated that long-range GABAergic cortico-striatal somatostatin-expressing neurons in the auditory cortex project to the dorsal striatum, and functionally inhibit the main projecting neuronal population, the spiny projecting neuron. Here we tested the hypothesis that parvalbumin-expressing neurons of the auditory cortex can also send long-range projections to the auditory striatum. To address this fundamental question, we took advantage of viral and non-viral anatomical tracing approaches to identify cortico-striatal parvalbumin neurons (**CS-Parv inhibitory projections → auditory striatum**). Here, we describe their anatomical distribution in the auditory cortex and determine the anatomical and electrophysiological properties of layer 5 CS-Parv neurons. We also analyzed their characteristic voltage-dependent membrane potential gamma oscillation, showing that intrinsic membrane mechanisms generate them. The inherent membrane mechanisms can also trigger intermittent and irregular bursts (stuttering) of the action potential in response to steps of depolarizing current pulses.

## Introduction

Since the late 19th century, the Spanish neuroanatomist Santiago Ramon y Cajal postulated the importance of interneurons in the neocortex (Ramon y Cajal et al., 1988; Benavides-Piccione and DeFelipe, 2007). After observing and studying the neuronal diversity and anatomy in several mammalian species, he concluded that “*the functional excellence of the human brain is intimately linked to the prodigious abundance and unwonted wealth of forms of the so-called neurons with short axon*” (i.e., GABAergic interneurons; Cajal, 1897). Since then, the local connectivity and neuronal computations of GABAergic “interneurons” in the cerebral cortex, which comprise 15–20% of the whole neuronal population (Xu et al., 2010; Rudy et al., 2011), has been extensively studied. This leading to the overall principle that excitation is both local and long-range, while inhibition is described as being exclusively local (for review see: Isaacson and Scanziani, 2011; Tremblay et al., 2016). The existence of long-range GABAergic neurons in rats, cats and monkey has been proven anatomically since the 80s (Seress and Ribak, 1983; Ribak et al., 1986; Toth and Freund, 1992; McDonald and Burkhalter, 1993; Toth et al., 1993; Freund and Buzsaki, 1996; Tomioka et al., 2005, 2015; Higo et al., 2007, 2009; Tomioka and Rockland, 2007; Tamamaki and Tomioka, 2010; for review see: Caputi et al., 2013; Tremblay et al., 2016). However, only recent investigation has been engaged to understand the functional significance of long-range GABAergic neurons and how different subtypes play distinct roles in cortical processing according to their differences in morphology, electrophysiology, molecular content, and synaptic connectivity patterns (Melzer et al., 2012, 2017; Lee et al., 2014; Rock et al., 2016, 2018; Zurita et al., 2018a; Bertero et al., 2019).

Evidence from our lab and others showed a direct inhibitory projection from the cortex to the striatum (Rock et al., 2016; Melzer et al., 2017), a basal ganglia area that is involved in the movement, reward-learning, and action selection behavior (Znamenskiy and Zador, 2013; Bahuguna et al., 2015; Xiong et al., 2015; Melzer et al., 2017; Guo et al., 2019). Melzer et al. (2017) demonstrated that locomotion could be modulated through direct stimulation of long-range GABAergic neuron terminals in the motor striatum. Moreover, Rock et al. (2016) found that long-range GABAergic somatostatin-expressing neurons in the auditory cortex control the spike timing/generation in both direct and indirect pathway spiny projection neurons of the auditory striatum. Overall this suggests that the balance of direct excitation/inhibition can promote the transformation of the acoustic signal into reward-learning and action-selection behaviors. However, no evidence of long-range GABAergic parvalbumin-expressing neurons from the auditory cortex to the auditory striatum has been shown yet. The present study focused on three main goals: (1) determine the laminar and areal distribution of cortico-striatal parvalbumin-expressing (CS-Parv neurons) neurons in the auditory cortex; (2) describe the anatomical and electrophysiological properties of these neurons; and (3) determine the impact that the voltage-dependent membrane potential resonance has on the spiking pattern of layer 5 CS-Parv neurons.

We addressed this fundamental question using both anterograde and retrograde anatomical methods in conjunction with *in vitro* electrophysiology. Using these techniques, we demonstrate, for the first time, the existence of parvalbumin-expressing GABAergic neurons in the auditory cortex with projections to the auditory striatum. We found that steps of depolarizing current pulses in layer 5 CS-Parv neurons can trigger intermittent and irregular bursts (stuttering) of action potentials. Also, our data suggest that while the first action potential of layer 5 CS-Parv neurons is triggered by an oscillation, whose frequency is in the gamma frequency, the second action potential was maintained by a different membrane mechanism. In sum, we describe a previously unknown long-range parvalbumin-expressing cortico-striatal projection (**CS-Parv inhibitory projections → auditory striatum**) that is engaged in cortico-striatal communication.

## Materials and Methods

All animal procedures were approved by the Institutional Animal Care and Use Committee at the University of Texas at San Antonio. Procedures followed animal welfare guidelines set by the National Institutes of Health. Mice used in this experiment were housed in a vivarium with a 12 h light/dark schedule and *ad libitum* access to mouse chow and water.

### Transgenic Mouse Lines

The following mouse lines were used in this study:

C57BL/6: (Charles river, strain code#027); Parv-Cre: B6.129P2-Pvalbtm1(cre)Arbr/J (The Jackson Laboratory, stock #017320); ROSA-tdTomato reporter: B6.CG.Gt(ROSA)26Sortm14 (CAG-tdTomato)Hze/J (The Jackson Laboratory, stock #007914); ROSA-eYFP reporter: B6.129X1-Gt(ROSA)26Sortm1(EYFP)Cos/J (The Jackson Laboratory, stock #006148); Parv-Cre homozygous mice were crossed with ROSA-tdTomato or ROSA-eYFP reporter homozygous mice to generate Parv-Cre/tdTomato and Parv-Cre/YFP parvalbumin-containing neurons expressing both Cre and tdTomato/eYFP lines, respectively.

### Viral Vectors

AAV1-CAG-FLEX-EGFP-WPRE, titer 3.1 × 10^13^ VG/ml (Addgene viral prep # 51502-AAV1).

### Stereotaxic Injections

#### Basic Surgical Procedures

As described in our previous studies (Rock and Apicella, 2015; Rock et al., 2016, 2018; Zurita et al., 2018a,b; Bertero et al., 2019), mice were initially anesthetized with isoflurane (3%; 1 L/min O_2_ flow) in preparation for the stereotaxic injections detailed in the next section. Mice were head-fixed on a stereotaxic frame (model 1900, Kopf Instruments) using non-rupture ear bars, and anesthesia was maintained at 1–1.5% isoflurane for the duration of the surgery. Injections were performed using a pressure injector (Nanoject III, Drummond Scientific) mounted on the stereotaxic frame and were delivered through a borosilicate glass injection pipette (Wiretrol II, Drummond Scientific) with a taper length of ~30 mm and a tip diameter of ~50 μm. The pipette remained in place for 5 min before to start injecting at 4 nl/min rate and was left in place for 5 min after the injection to avoid viral backflow along the injection tract. Both male and female mice, P35–P40 at the time of the infusion, were used in these experiments.

### Retrograde Labeling

CS-Parv neurons in the auditory cortex were retrogradely labeled by injecting 30 nl of Red Retrobeads (lumafluor) in the right striatum of C57BL/6 (*n* = 3 animals from 1 litter) or Parv-Cre/YFP (*n* = 3 animals from 1 litter). Stereotaxic coordinates: 1.4 mm posterior and 3.4 mm lateral to bregma at a depth of 2.8 mm below the surface of the brain. Mice were transcardially perfused 14–21 days post injections and brain fixed and sliced for immunofluorescence and antibody staining.

Thirty nanolitre of AAV1-flex-GFP were injected in the right striatum of Parv-Cre/tdTomato (*n* = 7 animals, from 4 litters). Stereotaxic coordinates: 1.4 mm posterior and 3.35 mm lateral to bregma at a depth of 2.8 mm below the surface of the brain. Mice were processed for electrophysiology 21–28 days post-injection.

### *In vitro* Slice Preparation and Recordings

As described in our previous studies (Rock and Apicella, 2015; Rock et al., 2016, 2018; Zurita et al., 2018a,b; Bertero et al., 2019), mice were anesthetized with isoflurane and decapitated. Coronal slices (300 μm) containing the area of interest were obtained on a vibratome (VT1200S, Leica) in a chilled cutting solution containing the following (in mM): 100 choline chloride, 25 NaHCO_3_, 25 D-glucose, 11.6 sodium ascorbate, 7 MgSO_4_ 3.1 sodium pyruvate, 2.5 KCl, 1.25 NaH_2_PO_4_, 0.5 CaCl_2_. Slices were then incubated in oxygenated artificial cerebrospinal fluid (ACSF) in a submerged chamber at 35–37°C for 30 min and then room temperature (21–25°C) until recordings were performed. ACSF contained the following (in mM): 126 NaCl, 26 NaHCO_3_, 10 D-glucose, 2.5 KCl, 2 CaCl_2_, 1.25 NaH_2_PO_4_, 1 MgCl_2_; osmolarity was ~290 Osm/L.

Whole-cell recordings were performed in 31–33°C ACSF. Thin-walled borosilicate glass pipettes (Warner Instruments) were pulled on a vertical pipette puller (PC-10, Narishige). They typically were in the range of 3–5 MΩ resistance. Intrinsic properties were recorded in the current-clamp configuration using a potassium-based intracellular solution which contained the following (in mM): 120 potassium gluconate, 20 KCl, 10 HEPES, 10 phosphocreatine, 4 ATP, 0.3 GTP, 0.2 EGTA, and 0.3–0.5% biocytin).

Signals were sampled at 10 kHz and filtered (lowpass filter) at 4 kHz. Pharmacological blockers used were as follows: CPP (5 μM; Tocris Bioscience), NBQX (10 μM; Abcam), and gabazine (25 μM; Abcam). Hardware control and data acquisition were performed with the Matlab-based Ephus package^1^ (Suter et al., 2010). The resting membrane potential (*V*_m_) was calculated in current-clamp mode (*I* = 0) immediately after breaking in. Series (*R*_s_) and input resistance (*R*_in_) were calculated in voltage-clamp mode (*V*_hold_ = −70 mV) by giving a −5 mV step, which resulted in transient current responses. *R*_s_ was determined by dividing the voltage step amplitude by the peak of the capacitive current generated by the voltage step. The difference between baseline and steady-state hyperpolarized current (Δ*I*) was used to calculate *R*_in_ using the following formula: *R*_in_ = −5 mV/Δ*I* − *R*_s_. Subthreshold and suprathreshold membrane responses in current-clamp were elicited by injecting −100 to +500 pA in 50 pA increments at V_rest_. The first resulting AP at rheobase (the minimal current of infinite duration (experimentally limited to 1 s) required to generate an AP) was analyzed for AP width. The adaptation ratio was measured at the current step that gave the closest APs firing rate to 20 Hz. The adaptation ratio was calculated, dividing the first instantaneous frequency by the last (f_2_/f_last_) or dividing the third instantaneous frequency by the fifth (f_3_/f_5_). Afterhyperpolarization (AHP) was calculated as the difference between the threshold and minimum membrane potential after an action potential. For the analysis of oscillations and action potential bursts, we used 20 s long steps of depolarizing current pulses. These current pulses were able to trigger intermittent and irregular bursts (stuttering) of action potential while holding the membrane potential at −70 mV. The analysis of membrane potential oscillations and relation with action potential generation was performed as described in Bracci et al. (2003). Briefly, the oscillation threshold was defined as the level of membrane potential at which spontaneous depolarizing and hyperpolarizing fluctuations of the membrane potential were larger than 1 mV. The cycle period of each oscillation was designated as the interval between two consecutive peaks. The inverse of the period was used to determine the frequency of the oscillation.

Spectral analysis of the oscillations was performed by using a Fast Fourier transform (FFT) custom MATLAB (MathWorks) routine. For oscillation analysis, *T_Osc.Peak2-Osc.Peak1_* was defined as the time interval between the last two consecutive peaks of oscillations before a burst of APs. *T_SpikeOsc.Peak2_* was defined as the interval between the last peak before an AP burst and the time when the last oscillation peak was higher than that of the preceding membrane potential. Oscillation slope (expressed in mV ms^−1^) was calculated as the slope of the line connecting an oscillation trough to the next oscillation peak. The pre-spike slope was defined as the slope of a line connecting the trough of the last oscillation before a spike burst and the point where the last peak oscillation amplitude was greater than the membrane potential amplitude. In each neuron analyzed a comparison between *T_Osc.Peak2-Osc.Peak1_* and *T_SpikeOsc.Peak2_* and between the oscillation slope and the pre-spike slope was performed by analyzing each of the pause and bursts obtained during 20 s of long of near-threshold current steps. In each cell, at least five spike bursts (preceded by oscillations) were used for analysis. The histogram of the values of *T_SpikeOsc.Peak2_*/*T_Osc.Peak2-Osc.Peak1_* and pre-spike slope/oscillation slope were obtained by calculating these ratios for each spike burst from different CS-Parv neurons.

### Immunohistochemistry and Histology

As described in our previous studies (Rock and Apicella, 2015; Rock et al., 2016, 2018; Zurita et al., 2018a,b; Bertero et al., 2019), mice were transcardially perfused with 4% paraformaldehyde, brains were dissected, postfixed overnight at 4°C, and coronal sections (100 μm thick) were obtained with a vibratome (VT1200S, Leica). Immunohisto-chemical procedures were performed on free-floating sections using: rabbit anti-GFP (for YFP, 1:500; Abcam, catalog #ab13970), rabbit anti-parvalbumin (1:1,000, Abcam, catalog #ab11427), primary antibodies, followed by AlexaFluor 633 goat anti-rabbit IgG (1:500; Life Technologies) and AlexaFluor 488 goat anti-chicken IgG (1:500; Abcam) secondary antibodies.

During whole-cell recordings, neurons were filled with an internal solution containing 0.3–0.5% biocytin. Filled neurons were held for at least 20 min, and then the slices were fixed in a formalin solution (neutral buffered, 10% solution; Sigma-Aldrich) for 1–7 days at 4°C. Fixed slices were then thoroughly washed in PBS, incubated overnight in a 4% streptavidin (AlexaFluor 680 conjugate; Life Technologies) solution, washed in PBS, and mounted with Fluoromount-G (Southern Biotech) on a glass microscope slide.

### Quantification of Laminar Distribution of CS-Parv Neurons in the AC

The mice, previously injected with a retrograde tracer into the auditory striatum (see “Materials and Methods” section above), were deeply anesthetized with 5% isoflurane, perfused, and the brain was fixed using the same procedures as previously described in Bertero et al. (2019). The fixed brain was then sectioned into 100 μm thick slices on a vibrating microtome. After washing in PBS, the slices were mounted on microscope slides, and images were taken with an Olympus SZX7 microscope. Images of 100 μm thick slices expressing the retrograde label GFP were rotated, cropped, and the brightness/contrast was adjusted in ImageJ. Using Adobe Illustrator, epifluorescence images were overlaid onto images from the Allen Mouse Brain Reference Atlas for coronal slices (Allen Institute for Brain Science) and aligned using anatomical landmarks such as the rhinal fissure and subcortical structures. Dorsal, primary, and ventral areas of the AC were identified using the overlaid reference images. A similar approach was used to determine the laminar distribution of the CS-Parv neurons. The distance from the pia to the white matter was normalized to 1,000 μm. Auditory cortex layer boundaries were determined as described in Ji et al. (2016) and Zurita et al. (2018b), as normalized depth from pia: layer 1 = 0–158 μm; layer 2/3 = 159–368 μm, layer 4 = 369–526 μm, layer 5 = 527–789 μm; layer 6 = 790–1,000 μm.

### Data Analysis

Figure error bars represent SEM. Data analysis was performed offline using custom MATLAB (MathWorks) routines. Group comparisons were made using the rank-sum test, and one-way *ANOVA*, with significance defined as *p < 0.05, and **p < 0.01.

## Results

### Anatomical Identification of CS-Parv Neurons in the Auditory Cortex

**Figure 1:**
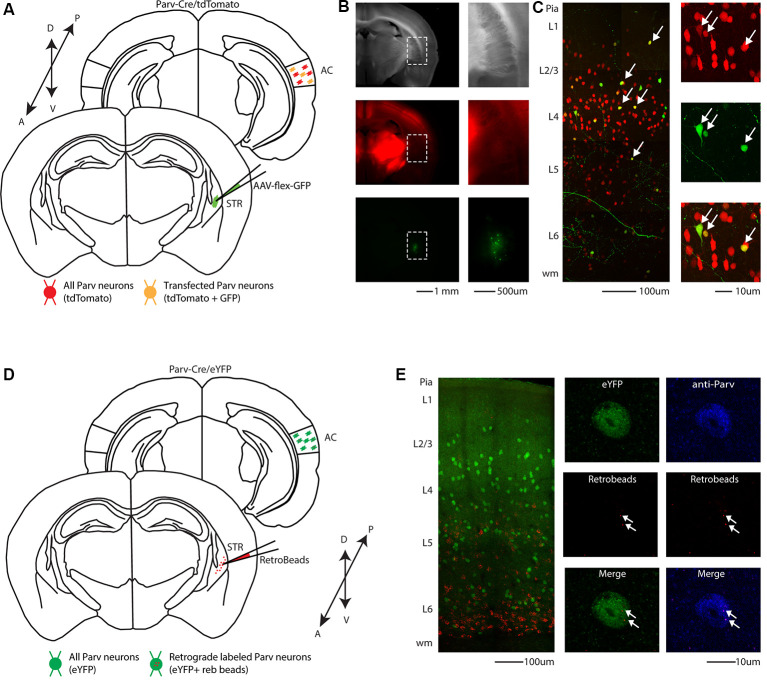
Cre-dependent identification of CS-Parv neurons in the mouse auditory cortex. **(A)** Schematic depicting injection site using the Parv-Cre-tdTomato transgenic mouse line to identify CS-Parv neurons in the auditory cortex. Bottom, striatum: green AAV1-Flex-GFP injection site; yellow Parv somata were coexpressing GFP and tdTomato; red Parv-tdTomato-positive interneurons. Top, auditory cortex: yellow CS-Parv somata coexpressing GFP and tdTomato; red Parv-tdTomato-positive “interneurons.” **(B)** Epifluorescence images of Parv tdTomato-positive neurons. Left, top: bright-field image of a slice containing the striatum injection site of AAV1-Flex-GFP in the Parv-Cre.tdTomato transgenic mouse line. Left, middle: tdTomato-expressing Parv neurons in the Parv-Cre-tdTomato transgenic mouse line. Left, bottom: GFP-positive Parv neurons in the dorsal striatum containing the viral injection of AAV1-Flex-GFP in the Parv-Cre-tdTomato transgenic mouse line. The dashed line indicates the striatum boundaries containing the striatum injection site of AAV1-Flex-GFP. Right, top: higher magnification of the bright-field image of a slice containing the striatum injection site of AAV1-Flex-GFP in the Parv-Cre.tdTomato transgenic mouse line. Right, middle: higher magnification image of tdTomato-expressing Parv neurons in the Parv-Cre-tdTomato transgenic mouse line. Right, bottom: higher magnification image of GFP-positive Parv neurons in dorsal striatum containing the viral injection of AAV1.GFP.Flex in the Parv-Cre-tdTomato transgenic mouse line. **(C)** Left: overlay image of GFP-positive Parv neurons in the auditory cortex identified by viral injection of AAV1.GFP.Flex and Parv neurons in the Parv-Cre-tdTomato transgenic mouse line. The dashed box and the arrows indicate the location of the somata of CS-Parv neurons. Top, right: tdTomato-expressing Parv neurons in the Parv-Cre-tdTomato transgenic mouse line. Middle, right: GFP-positive CS-Parv neurons in the auditory cortex retrogradely identified by viral injection of AAV1.GFP.Flex in the dorsal striatum of the Parv-Cre-tdTomato transgenic mouse line. Bottom right: overlay of GFP and tdTomato images. The arrow indicates the location of the CS-Parv neurons. **(D)** Schematic depicting the injection site of red RetroBeads using the Parv-Cre/eYFP transgenic mouse line to identify CS-Parv neurons in the auditory cortex. Bottom, striatum: red RetroBeads injection site; Top: auditory cortex: red beads retro-labeled CS-Parv neurons identified by YFP expression (green). **(E)** Left: overlay image of red-positive neurons in the auditory cortex identified by injection of retrograde beads in the dorsal striatum and Parv eYFP neurons in the Parv-Cre/eYFP transgenic mouse line. Middle: high magnification epifluorescence images of Parv red-beads-positive neurons. Middle, top: eYFP-positive Parv neurons in the auditory cortex in the Parv-Cre-tdTomato transgenic mouse line. Middle, center: CS-Parv neurons identified by anatomical retrograde labeling in the Parv-Cre-eYFP transgenic mouse line. Middle, bottom: overlay of eYFP and retrograde beads labeled CS-Parv neurons. The arrows indicate the location of the red beads in the CS-Parv neurons. Top, right: CS-Parv neurons immunostained with anti-Parv. Middle, right: CS-Parv neurons identified by anatomical retrograde labeling in the Parv-Cre-eYFP transgenic mouse line. Bottom, right: overlay of CS-Parv neurons immunostained with anti-parv and retrograde beads labeled CS-Parv neurons. The arrows indicate the location of the red beads in the CS-Parv neuron.

To visualize long-range GABAergic projection originating in the auditory cortex (AC) and terminating in the ipsilateral striatum, we conditionally expressed GFP in parvalbumin-expressing neurons (from this point forward referred to as CS-Parv neurons) by injecting AAV1-Flex-GFP into the right striatum of Parv-Cre/tdTomato transgenic mice (animals: *n* = 7; litter *n* = 6; Figure 1). GFP was colocalized with Parv/tdTomato-expressing neurons in the AC (Figures 1B,C). We used this viral approach because data from our study and others (Rothermel et al., 2013; Rock et al., 2016, 2018; Zurita et al., 2018a; Bertero et al., 2019) demonstrated that AAV1-Flex. Flex viral vectors exhibited both anterograde and retrograde transfection capabilities. From the center of the injection, the spread of the viral tracer was estimated at around 600 μm anteroposterior, and 900 μm dorsal-ventral/medial-lateral, with negligible or no evidence of tracer spillover in the somatosensory cortex above the injection site (Figure 1B). This approach allowed us to identify transfected somata in the ipsilateral AC and to highlight long-range cortico-striatal parvalbumin-expressing neurons (Figure 1C).

**Figure 2:**
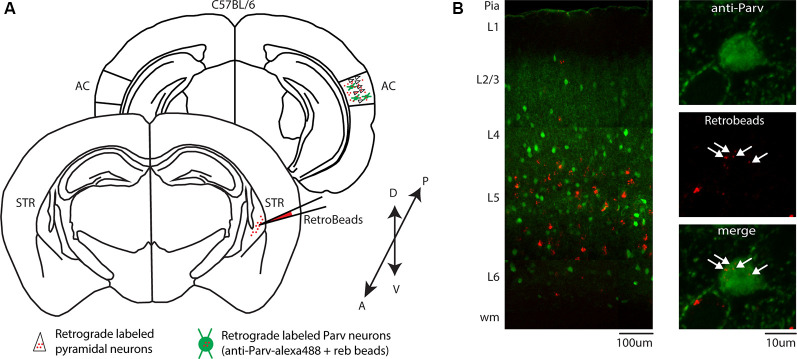
Identification of CS-Parv neurons in the auditory cortex of wild-type C57BL/6 mice. **(A)** Schematic depicting the injection site of red RetroBeads using the wild-type C57BL/6 mouse line to identify CS-Parv neurons in the auditory cortex. Bottom, striatum: red RetroBeads injection site; Top, auditory cortex: red beads retrolabeled pyramidal and CS-Parv neurons identified by immunostaining with anti-Parv. **(B)** Left: overlay image of red-positive retrograde labeled neurons in the auditory cortex identified by injection of retrograde beads in the dorsal striatum and anti-Parv in the wild-type C57BL/6 mouse line. Top, right: anti-Parv immunostained Parv neurons in the auditory cortex in the wild-type C57BL/6 mouse line. Middle, right: CS-Parv neurons identified by anatomical retrograde labeling in the wild-type C57BL/6 mouse line. The arrow indicates the location of the red beads in the CS-Parv neurons. Bottom, right: overlay of CS-Parv neurons immunostained with anti-Parv and retrograde beads labeled CS-Parv neurons. The arrow indicates the location of the red beads in the CS-Parv neurons.

In a different set of experiments, to visualize long-range parvalbumin projections originating in the AC and terminating in the striatum, we injected a well-established non-viral retrograde tracer, such as red Retrobeads, into the right auditory striatum of Parv-Cre/eYFP transgenic mice (animals: *n* = 3; litters: *n* = 1) and analyzed retrogradely labeled neurons in the AC (Figure 1D). The injection site was centered primarily in the posterior region of the dorsal striatum. From the center of the injection, the spread of the tracer was estimated at around 500 μm anteroposterior, 900 μm dorsal-ventral, and 500 μm medial-lateral. For all our injections (animals: *n* = 3; litters: *n* = 1), there was no evidence and/or negligible of tracer spillover or deposit in the somatosensory cortex above the injection site. Using this method, we found that, in CS-Parv neurons’ thin optical slices (1 μm thick optical slices, 1–3 slices z projection), YFP was colocalized with red RetroBeads of the AC in the hemisphere ipsilateral to the injection site (Figure 1E, middle panels). We further characterized the CS-Parv neurons by confirming their expression of the calcium-binding protein parvalbumin (Parv; Figure 1E, right panels). To further corroborate our findings, we injected wild type C57BL/6 mice (animals: *n* = 3; litter *n* = 1), the genetic background of Parv-Cre, and derived reporter lines, with red Retrobeads in the right striatum (Figure 2A). Again, we were able to confirm the presence of double-positive Parv/Red beads neurons in the hemisphere of the AC ipsilateral to the injection site (Figure 2B). Our results demonstrate that, independently of the mouse strain and reporter used, long-range CS-Parv neurons can be detected in the AC with well-established retrograde tracing techniques. The red beads retrogradely methods further validated our viral retrograde approach, which exhibits high labeling efficiency of CS-Parv neurons in the auditory cortex of injected mice.

### Areal and Laminar Characterization of CS-Parv Neurons in the Auditory Cortex

**Figure 3:**
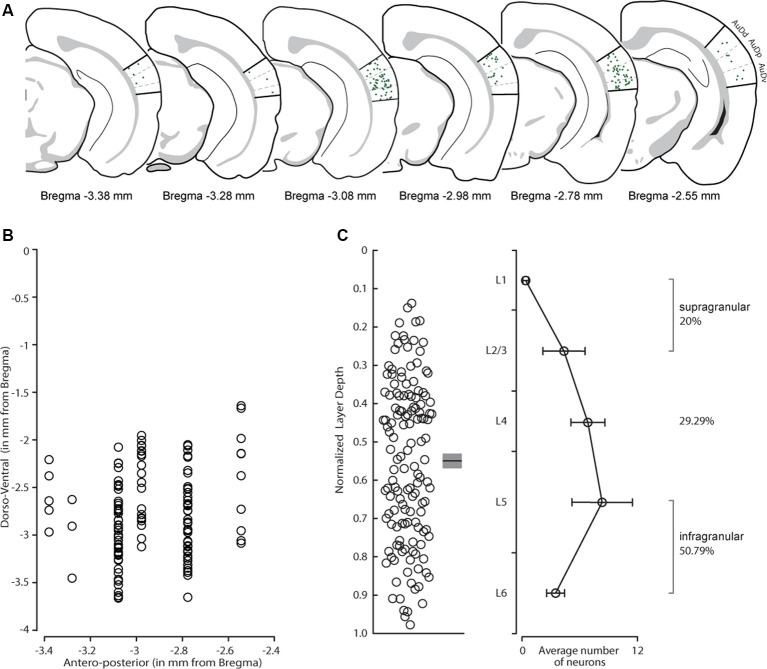
Distribution of CS-Parv neurons in the mouse auditory cortex. **(A)** Anatomical distribution of CS-Parv neurons in serial coronal sections. **(B)** The plot shows the anteroposterior and dorso-ventral soma location of CS-Parv neurons. **(C)** Left: plot shows the group average soma location (±SEM) of CS-Parv neurons. The black circles mark the absolute distances from the pia to the soma (CS-Parv: *n* = 140 neurons, *n* = 6 animals). Right: laminar distribution of CS-Parv neurons. For the quantification of SC-Parv neurons coexpressing GFP and tdTomato, the distance between the pia and white matter was normalized to 1,000 μm, and the cortex was divided into layers based on the following thicknesses: L1, 0–158 μm; L2/3, 159–368 μm; L4, 369–526 μm; L5, 501–750 μm; and L6, 790–1,000 μm. Average neuron number per layer was calculated among *n* = 6 animals: L1, 0.33 ± 0.30 neurons; L2/3, 4.33 ± 2.19 neurons; L4, 6.83 ± 1.77 neurons; L5, 8.33 ± 3.15 neurons; L6, 3.5 ± 0.94 neurons.

We first determined the areal distribution of CS-Parv neurons. Double labeled tdtomato/GFP neurons were observed in both the dorsal-ventral and anteroposterior extent of the AC, including the dorsal, primary, and ventral auditory cortex (Figure 3A), as quantified in Figure 3B (*n* = 6 animals, *n* = 9 slices, 300 μm thick). The distribution of the retrogradely labeled neurons is indicated in Figure 3 by overlapping coronal epifluorescence images with reference images from the online mouse atlas provided by the Allen Institute for Brain Science^2^ (coronal atlas). Next, we determine the laminar distribution of the CS-Parv neurons in the AC. Using the same method described above, we found that cortical CS-Parv neurons were spanning all layers of the AC in the hemisphere ipsilateral to the injection site (Figure 3C: *n* = 140 neurons, six animals from 5 litters, average depth 0.549 ± 0.018 mm from pia). Interestingly, as already shown for the auditory cortical excitatory projections (Znamenskiy and Zador, 2013), the majority (50.79%; Layer 5 and 6 CS-Parv neurons: *n* = 71/140 neurons) of the long-range CS-Parv neurons were located in the infragranular region of the AC (L1, 0.33 ± 0.30 neurons; L2/3, 4.33 ± 2.19 neurons; L4, 6.83 ± 1.77 neurons; L5, 8.33 ± 3.15 neurons; L6, 3.5 ± 0.94 neurons). These multiple complementary data sets confirm that, in the entire AC, long-range CS-Parv GABAergic neurons send a direct projection to the posterior region of the dorsal striatum (**CS-Parv inhibitory projections → striatum**).

### Morphological and Electrophysiological Characterization of Layer 5 CS-Parv Neurons

Given the higher abundance of CS-Parv in layer 5 of the auditory cortex, and the high heterogeneity of GABAergic neurons, especially across different cortical layers, we decided to focus our study on layer 5 CS-Parv neurons that were retrogradely labeled by injecting the AAV1-Flex-GFP virus in the right striatum. This approach allowed us to visually identify and record from layer 5 CS-Parv neurons using whole-cell patch-clamp (Figure 1C).

**Figure 4:**
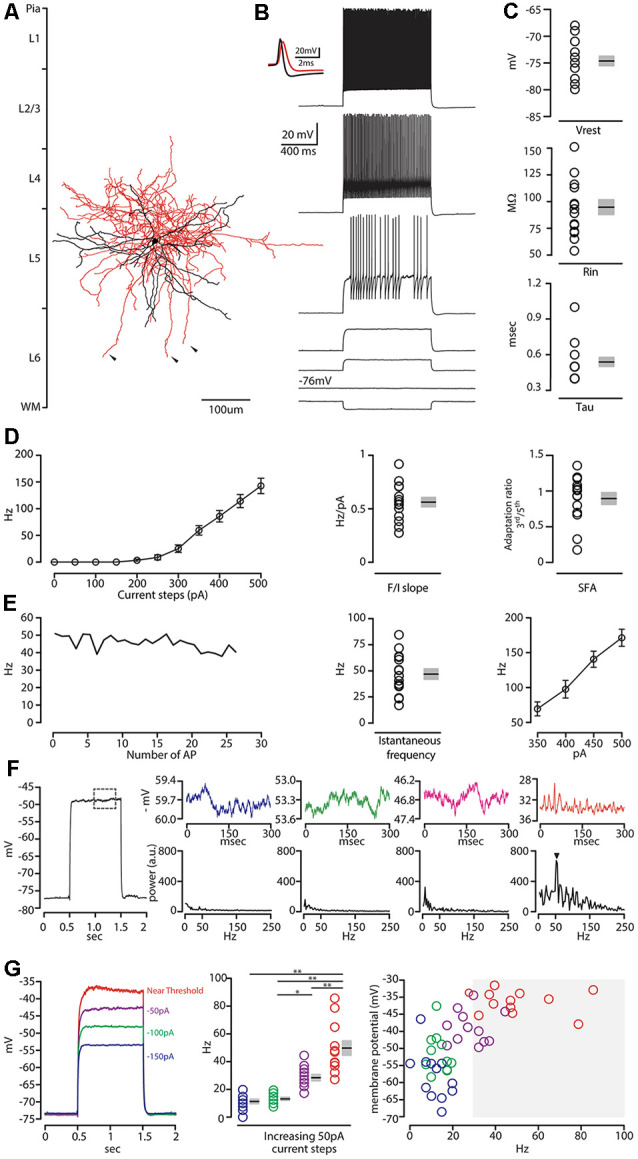
Morphological and electrophysiological characterization of layer 5 CS-Parv neurons. **(A)** Morphological reconstruction of one CS-Parv neuron (dendrites, black; axons, red). **(B)** Train of action potentials recorded in a layer 5 GFP/tdTomato-positive CS-Parv neuron during step current injection (1.0 s, 100 pA pulse). Top left inset, single action potential from layer 5 GFP/tdTomato-positive CS-Parv neuron (black); compare to an action potential from a corticostriatal somatostatin-expressing GABAergic neuron (red). **(C)** Top: summary plot of V_rest_: resting membrane potential; Middle: R_i_: input resistance; Bottom: Tau: membrane time constant; from CS-Parv neurons (neurons *n* = 15, animals *n* = 8; including group average ± SEM). **(D)** Left: summary plot of averaging firing rate per current step amplitude recorded from layer 5 CS-Parv neurons (black circles, *n* = 15 neurons, animals *n* = 8), including group averages (± SEM). Middle: same as in panel **(D)** for F/I slope. Right: same as in panel **(D)**, for *spike frequency adaptation (SFA; f*3rd/*f*5th). **(E)** Left: representative instantaneous frequency near-threshold as a function of the number of AP from layer 5 CS-Parv. Middle: summary plot of instantaneous frequency near-threshold from layer 5 CS-Parv neurons (black circles, *n* = 15 neurons, animals *n* = 8), including group averages (± SEM). Right: summary plot of instantaneous firing frequency in response to increasing depolarizing current (350–500 pA, 50 pA increments) for layer 5 CS-Parv neurons (black circles, *n* = 15 neurons, animals *n* = 8) including group averages (± SEM). **(F)** Left: example trace in response to depolarizing 1 s current. The dashed box represents the region analyzed in the left section of the panel. Top row: representative membrane potential oscillation at four increasing 50 pA increasing current steps to reach near-threshold membrane potential (blue trace: −150 pA; green trace: −100 pA; magenta trace: −50 pA of the near-threshold current; red trace: membrane potential near-threshold defined as 0 pA current injection from near-threshold). Bottom row: corresponding frequency contents of the four different membrane potentials. The arrow indicates the peak in the gamma range (52 Hz) of membrane oscillation at the near-threshold potential. **(G)** Left: representative membrane potential changes in response to 1 s long 50 pA increasing current steps (blue, green, and purple traces) to reach near-threshold (red trace). Middle: frequency content of membrane oscillation in response to increasing current steps, color code as in panel **(G)** (left, *n* = 12 neurons, including group average ± SEM, *p < 0.05; **p < 0.001). Right: membrane potential in response to increasing current steps [color code as in panel **(G)** (left as a function of the frequency content of membrane oscillation]. Gray box: gamma frequency range (30–100 Hz, *n* = 12 neurons).

**Figure 5:**
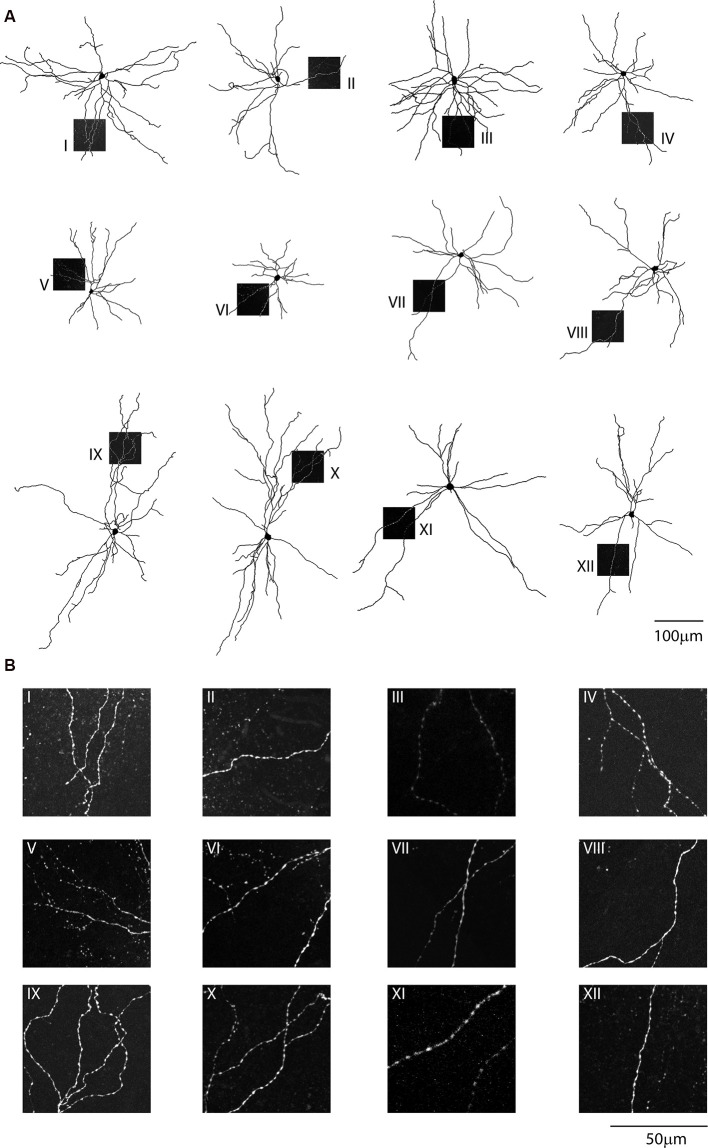
Single-cell reconstruction of the dendritic arborization of biocytin-filled retrograde labeled CS-Parv neurons. **(A)** All neurons are oriented towards pia and the dashed boxes indicate the location of the dendritic confocal images for each neuron. **(B)** Each neuron displays no dendritic spines, as shown in the corresponding high-resolution confocal images.

Confocal images of biocytin filled layer 5 CS-Parv neurons (12 out of 15 recorded neurons) showed that they are similar to Parv basket-like GABAergic neurons in their cortical axonal morphology (Figure 4A), aspiny and multipolar dendritic arbor (Figure 5), and send collaterals towards the subcortical white matter. In addition to the basket-like axonal morphology, a small fraction (4 out of 15 recorded neurons) exhibit a translaminar axonal distribution (data not shown). Electrophysiological characterization (*n* = 15 neurons, animals *n* = 8, litters *n* = 5) showed that layer 5 CS-Parv neurons display common fast-spiking properties, including a high rheobase (the smallest current step evoking an action potential: 306.7 ± 20.04 pA), a narrower action potential compared to somatostatin-expressing GABAergic neurons in the AC (Figure 4B, inset, showing a representative layer 5 CS-Parv action potential in black and a representative somatostatin-expressing neuron action potential in red; layer 5 CS-Parv action potential half-width: 0.26 ± 0.01 ms), large fast afterhyperpolarization (fAHP: −17.6 ± 0.6 mV), and no synaptic integration (data not shown; Table 1). Basic electrophysiology properties include: resting membrane potential, −74.7 ± 0.89 mV; input resistance, 96.4 ± 6.8 MΩ; membrane time constant, 0.54 ± 0.04 ms (Figure 4C). The sustained current injection also showed that the majority of layer 5 CS-Parv neurons exhibit a suprathreshold stuttering firing pattern (Li and Huntsman, 2014), and continuous firing of action potential under a further increase of current injection that can reach up to 150 Hz (F/I summary plot: F/I slope: 0.56 ± 0.04 Hz/pA; Figure 4D, left and middle panels) with no spike frequency adaptation (SFA ratio: 0.97 ± 0.03 third/fifth, 0.84 ± 0.04 s/last; Figure 4D, right panel). Near-threshold, the initial instantaneous frequency was 47.16 ± 4.95 Hz, remained constant in time and increased in responses to higher depolarizing current steps (Figure 4E). Further characterization of membrane potential in response to depolarizing currents also showed that the frequency content of membrane oscillations shifted towards the gamma band with increasing steps of depolarizing current pulses (see representative membrane potential oscillations and frequency content analysis in Figure 4F). We, therefore, analyzed the frequency content for each cell to the near-threshold current steps and three consecutive steps of less depolarizing current pulses (minus 50 pA each, Figure 4G). This allowed us to demonstrate that the gamma band frequency content of membrane oscillation was correlated to the membrane potential increase. As shown in Figure 4G, no gamma oscillations are detected up to 100 pA below threshold, and the gamma content significantly increased at 50 pA below threshold (column factor *p* = 1.36 × 10^−11^, *f* = 34.32 one-way *ANOVA*; multiple comparisons: step 1 vs. step 2 *p* = 0.97, step 2 vs. step 3 *p* = 0.0014, step 3 vs. step 4 *p* < 0.00001, step 2 vs. step 3 *p* = 0.005, step 2 vs. step 4 *p* < 0.00001, step 3 vs. step 4 *p* = 0.0001). This increase in gamma-range oscillation was also correlated to the increase in membrane potential (Figure 4G). These data show that layer 5 CS-Parv neuronal morphology and intrinsic electrophysiological properties resemble those of the Parv basket-like “interneurons.”

**Table 1:** Layer 5 cortico-striatal Parvalbumin neurons intrinsic properties.

Parameter	Mean ± SEM
Resting potential (mV)	−74.66 ± 0.88
Input resistance (MΩ)	96.4 ± 6.77
Membrane time constant Tau (ms)	0.54 ± 0.03
Rheobase (pA)	306.66 ± 20.03
After hyperpolarization (mV)	−17.60 ± 0.64
After depolarization (mV)	14.011 ± 0.57
Action potential threshold (mV)	−33.58 ± 1.34
Action potential height (mV)	43.20 ± 1.96
Action potential halfwidth (ms)	0.25 ± 0.01
F/I slope (Hz/pA)	0.56 ± 0.04
Spike frequency adaptation (third/fifth)	0.96 ± 0.03
Spike frequency adaptation (second/last)	0.84 ± 0.04

### Relationship Between Membrane Near-Threshold Oscillations and Action Potentials

**Figure 6:**
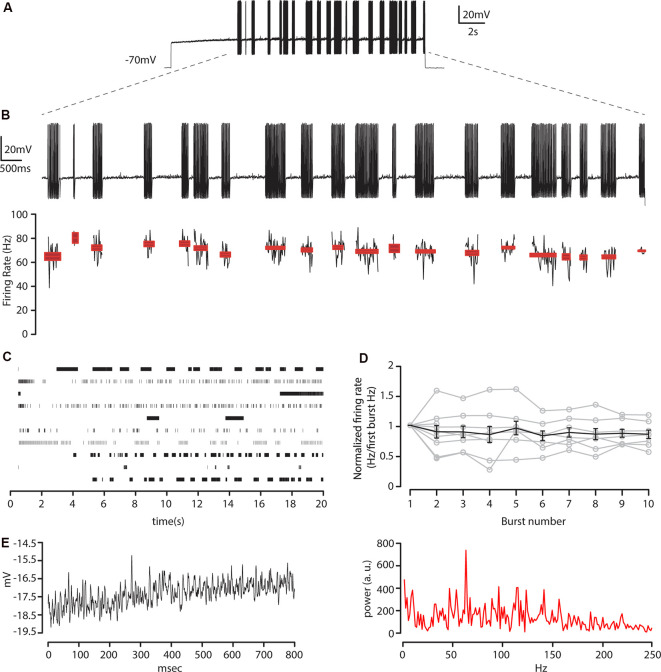
Membrane resonance in response to near-threshold sustained current steps. **(A)** Train of action potentials recorded at −70 mV from layer 5 CS-Parv neurons in response to a step of depolarizing current pulse (20 s, pA = near-threshold). **(B)** Top: enlargement of bursts in **(A)**. Bottom: instantaneous firing rate for each burst and spike count for each burst, including average ± SEM **(C)** Raster plot for each neuron recorded in response to steps of depolarizing current pulses near-threshold (*n* = 10 neurons). **(D)** The average plot of firing rate for the first 10 bursts normalized to the average firing rate of the first burst of action potentials (gray traces: *n* = 11 neurons), including average ± SEM (black trace). **(E)** Left: an example of a membrane potential trajectory between bursts of action potentials. Right: corresponding frequency spectrum of the near-threshold oscillations during the pause. The peak of the spectrum corresponds to the gamma-band frequency (63.2 Hz).

We then characterized the relationship between membrane oscillations and action potentials train generation at near-threshold current steps. Since standard 1 s depolarizing current pulses were not suitable to describe the firing pattern of layer 5 CS-Parv neurons, we studied their (*n* = 11 neurons) response to long (20 s) current pulses, which evoked sequences of bursts (stuttering) and pauses (Figure 6A).

The striking feature of the layer 5 CS-Parv neurons was that their firing pattern during long positive current steps was mostly unpredictable. A typical profile of the layer 5 CS-Parv neurons behavior and instantaneous firing rate during these repeated bursts is shown in Figures 6A,B. The duration of the action potential bursts and the pauses varied from 100 ms to up to 10 s when a near-threshold current was injected at the same resting membrane potential, reaching up to 193 spikes per burst and ranging from 16.8 Hz to 101.6 Hz spiking rate, in a visible random fashion (Figure 6C). Before the first burst, two out of 10 neurons showed an initial single action potential at the start of the pulse, followed by a long pause. In comparison, three out of 10 neurons displayed a long delay before the first burst with no previous action potential. High-frequency repetitive firing following this pause exhibited a non-adaptation through the entire duration of the burst. The instantaneous firing frequency of each burst was nearly constant across them, as shown in Figure 6D in which the firing rate of the first 10 bursts was normalized on the first burst firing rate and, on average, no difference was found between the first and subsequent bursts firing rate (*p* = 0.9705, *f* = 0.31 one-way *ANOVA*).

In the period between the bursts, the membrane potential showed a near-threshold oscillation in the gamma frequency range (Figure 6E). The amplitude of these oscillations varied between 1 and 4 mV, and the frequency range was voltage-dependent and was completely absent at the resting membrane potential (data not shown). The presence of synaptic blockers during our recording [NBQX (10 μM), CPP (5 μM), and gabazine (25 μM)] and the observation that the oscillation frequencies are voltage-dependent suggested that this phenomenon is generated by intrinsic properties of the layer 5 CS-Parv neurons and was not preferentially reliant on the cortical connectivity patterns of these neurons.

**Figure 7:**
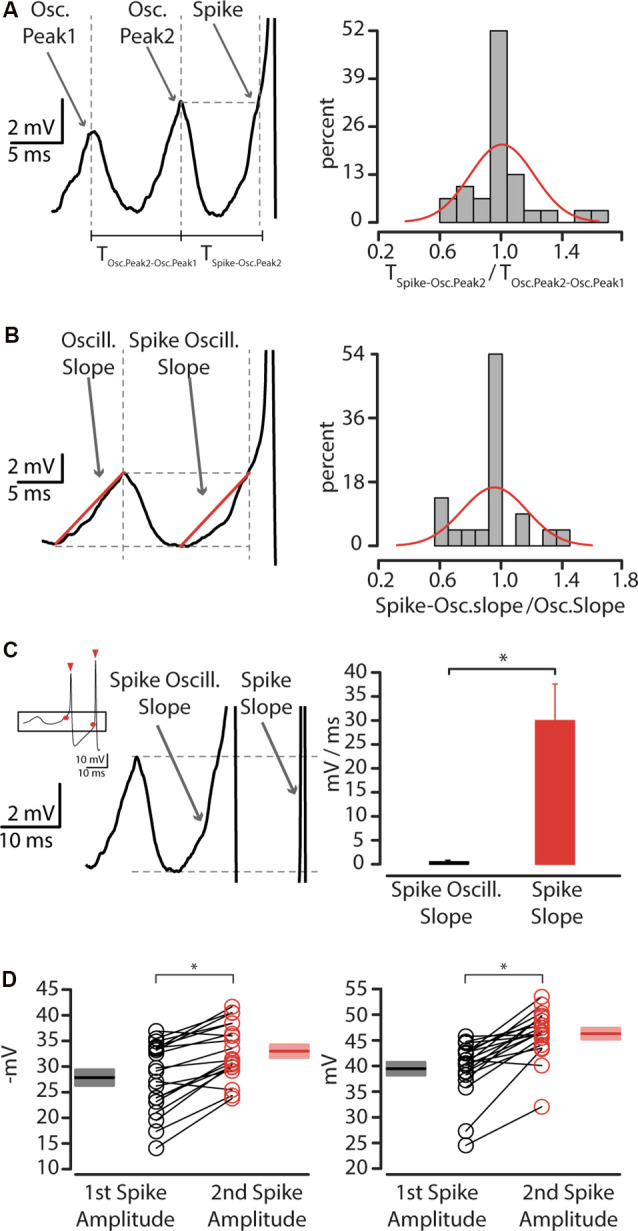
The relation between membrane oscillations and action potentials. **(A)** Left: the interpeak interval between oscillations preceding action potential. Right: quantification of the ratio between the interpeak interval of the last oscillation peak and spike, and the interpeak interval between second to last and last oscillation peak before an action potential, showing unitary distribution (mu = 1.007, sigma = 0.210). **(B)** Left: oscillation slope and spike-oscillation slope before first action potential. Right: quantification of the ratio between spike-oscillation slope and oscillation slope, showing unitary distribution (mu = 0.953, sigma = 0.213). **(C)** Left: spike-oscillation slope compared to spike slope. The inset shows a representative trace of action potentials thresholds (red circle) and amplitude (red triangles) Right: summary plot of the spike-oscillation slope, expressed in mV/ms ± SEM, *p < 0.05. **(D)** Left: plot of first (*n* = 35, black circles) and second (*n* = 35, red circles) AP threshold recorded from layer 5 CS-Parv neurons, including group average (±SEM, *p < 0.05). Right: Plot of first (*n* = 35, black circles) and second (*n* = 35, red circles) AP amplitude recorded from layer 5 CS-Parv neurons, including group average (±SEM, *p < 0.05).

We then investigated the relationship between layer 5 CS-Parv neurons oscillations and action potential (AP) generation. One clear aspect in the sequence of bursts and membrane potential oscillations was that the first spike of the burst was almost consistently preceded by oscillations and appeared to be triggered by the depolarizing phase of the oscillations. To test this quantitively, we used the same method used by Bracci et al. (2003). The Bracci’s method allowed us to investigate: (1) if the depolarizing membrane potential between the one that preceded the first spike of the burst with the preceding oscillation had a similar phase relationship; and (2) if the slope of the rising phase between them was comparable. To test the first point, we quantified the time interval between three consecutive oscillation peaks. *T_Osc.Peak2-Osc.Peak1_* was defined as the time interval between the last two consecutive oscillation peaks before a burst of action potentials (Figure 7A); and *T_SpikeOsc.Peak2_* as the interval between the last peak before an action potential burst and the time when the last oscillation peak amplitude was greater than the membrane potential amplitude (Figure 7A). As illustrated in Figure 7A, this allowed us to quantify the two-time intervals between the three oscillation peaks (T_Osc.Peak2-Osc.Peak1_; T_Spike-Osc.Peak2_).

The values of T_Spike-Osc.Peak2_ were not statistically significant form T_Osc.Peak2-Osc.Peak1_ (rank-sum test, *p* = 0.9), as shown by the summary histogram (*n* = 11 neurons), and their ratio was distributed around unity (Summary plot: Figure 7A, right). This suggests that the first AP of the burst was triggered by near-threshold oscillation potential that has the same peak-time intervals. To test the second point, we compared the slope of the depolarizing phase of the spike oscillation to that of the oscillation preceding the spike (Figure 7B, left). Again, the ratio of the spike and the oscillation slope was distributed around unity (Summary plot: Figure 7B, right). The slope data are consistent with the idea that the spike oscillation and the oscillation preceding the spike are characterized by the same kinetics. Altogether, these data suggest that the first action potential of the burst in the layer 5 CS-Parv neurons is caused by an oscillation in the gamma range that was sufficiently large to take the neuron above the firing threshold. Our data also indicated that the spike frequency was always higher than the near-threshold oscillation frequency (data not shown), suggesting that the second action potential of the burst is not triggered by the same oscillatory mechanism of the first action potential in the burst. To test this hypothesis, we compared the slope of the first and second action potential (Figure 7C). The summary plot shows that the slope of the second action potential is statistically faster than the first action potential (first spike slope: 0.52 ± 0.04 mV/ms; second spike slope: 29.95 ± 7.62 mV/ms; *n* = 11, rank-sum test, *p* = 2.1 × 10^−4^). The second actional potential differed from the first action potential also in the membrane threshold (first spike threshold: −27.84 ± 1.54 mV; second spike threshold: −32.99 ± 1.25 mV; rank-sum test, *p* = 0.0265) and amplitude (first spike amplitude: 39.46 ± 1.25 mV; second spike amplitude: 46.29 ± 1.08 mV; rank-sum test, 3.8 × 10^−5^; Figure 7D).

These data suggest that while the first AP of the layer 5 CS-Parv neuron burst is triggered by an oscillation, whose frequency is in the gamma range, the second action potential was maintained by a potential different membrane mechanism.

## Discussion

**Figure 8:**
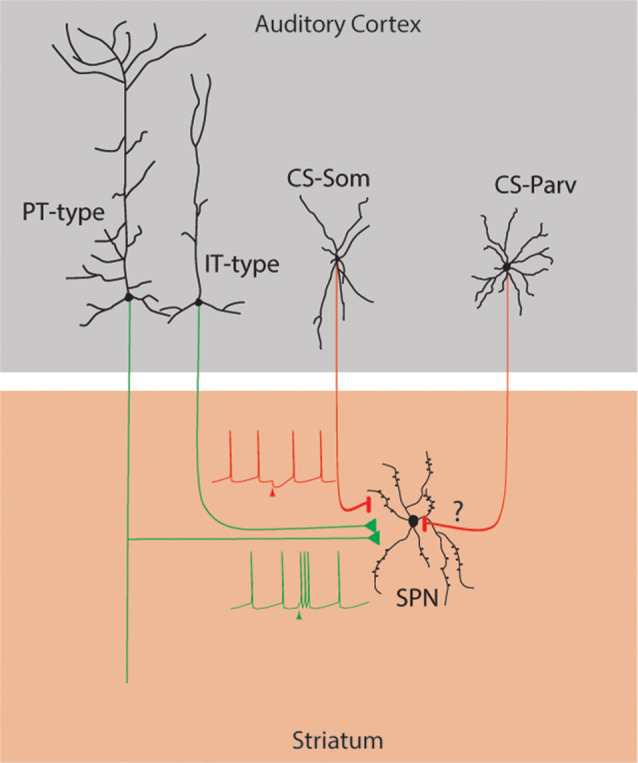
Summary diagram: Long-range cortico-striatal CS-Parv neurons. Green lines: excitatory inputs from intratelencephalic (IT-type) and projecting-type (PT-type) layer 5 pyramidal neurons. Red lines: inhibitory input from long-range GABAergic CS-Som and CS-Parv neurons.

In this study, we tested the hypothesis that parvalbumin-expressing neurons in the AC project to the ipsilateral dorsal striatum. Here, we identify a previously unknown auditory cortico-striatal long-range parvalbumin-expressing projection (**CS-Parv inhibitory projections → auditory striatum**; Figure 8) with two complementary labeling techniques and in different mouse lines.

As we described previously (Rock et al., 2016, 2018; Zurita et al., 2018a; Bertero et al., 2019), the employed viral and non-viral labeling approaches (restricted injection volume (focal injection) and variability in transfection leading to incomplete coverage of the cortical region) prevents us from determining the absolute number of CS-Parv neurons. Despite these caveats, our viral retrograde labeling approach allowed us to routinely determine the cortical distribution, axonal morphology, and electrophysiological properties of layer 5 long-range CS-Parv neurons and establish the presence of voltage-dependent membrane potential oscillations. We also showed that the layer distribution of CS-Parv neurons, with higher concentrations in the infragranular layer, is not reflecting the overall Parv neurons distribution, which generally shows higher abundancy in layer IV (Tremblay et al., 2016), paralleling the distribution of excitatory auditory cortico-striatal projections (Znamenskiy and Zador, 2013).

### Layer 5 CS-Parv Neurons: From Anatomical-Electrophysiological Properties to Circuits

Previous data from our lab highlighted that another class of long-range parvalbumin-expressing neurons projecting from the left auditory cortex to the contralateral one (corticocortical-Parv, CC-Parv neurons) can be distinguished from local Parv neurons based on their intrinsic properties (Zurita et al., 2018a). In particular, layer 5 CC-Parv neurons are characterized by a lower input resistance, a higher rheobase, a larger AHP, and lower instantaneous frequency at threshold compared to layer 5 non-callosal projecting Parv neurons (Zurita et al., 2018a), all features comparable to the hereby described layer 5 CS-Parv neurons. This observation lead to ask whether long-range Parv neurons sharing common electrophysiological properties can also share their projection pattern (i.e., can CC-Parv neurons also project to the ipsilateral striatum?) or constitute two segregate classes of long-range inhibitory neurons (i.e., are CC-Parv neurons only engaged in cortico-cortical circuitry while CS-Parv neurons only in corticofugal circuits)? These are only a few of the numerous questions that still need to be addressed concerning long-range GABAergic circuits in the neocortex. A crucial line of investigation will be to characterize how CS-Parv neurons may differ from local parvalbumin-expressing “short-axons interneurons.” For example, are the excitatory and inhibitory connectivity patterns of CS-Parv neurons similar and/or different to those with local short-axons parvalbumin-expressing “interneurons” (Xu and Callaway, 2009; Apicella et al., 2012; Pfeffer and Beltramo, 2017)? Do CS-Parv neurons modulate network oscillation (Buzsáki and Draguhn, 2004; Buzsáki and Wang, 2012)? Do CS-Parv neurons have different embryonic development as already demonstrated for the long-range GABAergic hippocampal neurons (Picardo et al., 2011; Christenson Wick et al., 2019)? Do CS-Parv neurons have different gene expression profiles (for review see Huang and Paul, 2019)? Do CS-Parv neurons form depressing inhibitory synapses as reported for parvalbumin-expressing basket-like “interneurons” (Reyes et al., 1998; Silberberg, 2008)? Does electrical synapse couple CS-Parv neurons, and if so, are they only electrically-coupled with the neurons expressing the similar molecular markers (for review see Connors, 2017)? Do CS-Parv co-release GABA and other neurotransmitters (Burnstock, 1976)? Do cortico-striatal long-range GABAergic neurons modulate cognition and behavior? Further studies will be fundamental to address all these numerous questions regarding long-range GABAergic circuits in the cortex.

### Layer 5 CS-Parv Neurons: From Circuits to Auditory Response Selectivity

Our results on layer 5 CS-Parv neurons demonstrate that these neurons morphologically resembled basket-like neurons, with massive axonal arborization in layer 5 that can span through layer IV and VI, and send collaterals towards the white matter and subcortical target(s). This suggests that layer 5 CS-Parv neurons are not only involved in long-range corticofugal circuits (together with excitatory neurons) but can also be embedded in local circuits involved in cortical sound processing as their short-range counterparts (Sun et al., 2013). Previous studies have shown that parvalbumin-expressing neurons receive input from different pathways (Helmstaedter et al., 2009; Xu and Callaway, 2009; Bagnall et al., 2011; Kubota et al., 2011; Tukker et al., 2013; Pfeffer and Beltramo, 2017), and have examined the dynamics of the membrane potential and mapped the sensory space of GABAergic “interneurons” (Niell and Stryker, 2008; Liu et al., 2009, 2010; Gentet et al., 2010; Kerlin et al., 2010; Runyan et al., 2010; Runyan and Sur, 2013; Li et al., 2015; Resnik and Polley, 2017) describing the existence of neurons that are characterized by broad and high tuned responses.

Also, studies in both motor and sensory cortex have characterized different connectivity patterns between subtypes of layer 5 pyramidal neurons and GABAergic “interneurons” (Markram, 1997; Morishima and Kawaguchi, 2006; Brown and Hestrin, 2009a,b; Dani and Nelson, 2009; Morishima et al., 2011; Apicella et al., 2012). Particularly, Sakata and Harris (2009) observed that, during a sound presentation, layer 5 thick-tufted neurons (such as layer 5 cortico-striatal excitatory/glutamatergic projecting-type (PT-type) neurons) received weaker inhibition than slender neurons [such as layer 5 cortico-striatal excitatory/glutamatergic intratelencephalic-type (IT-type) neurons]. More recently, Sun et al. (2013) suggested that intrinsic bursting pyramidal neurons (such as PT-type cortico-striatal neurons) have mechanisms of synaptic integration that are broader than pyramidal neurons with regular spiking properties (such as IT-type cortico-striatal neurons). Moreover, Sun et al. (2013) speculated the parvalbumin-expressing neurons are capable of providing feedforward inhibitory input preferentially to the PT-type neurons.

In this view, our finding of the existence of layer 5 CS-Parv neurons requires further experiments focused to better characterize the local synaptic connectivity partners of long-range CS-Parv neurons, and their activity in response to sensory stimuli. This will be crucial for the understanding of connectivity patterns of these neurons and their role in cortical auditory processing.

### Layer 5 CS-Parv Neurons: Cortical Oscillations

The anatomical and electrophysiological properties underlying different cell-types of long-range GABAergic neurons, as well as their connectivity patterns within the cortex is virtually unknown. A crucial goal is to determine the differences between CS-Parv neurons and local parvalbumin-expressing “short-axons interneurons” in the generation of locally cortical oscillations and the propagation along the cortico-striatal pathway. Cortical rhythms are a well-established feature of cortical neuronal activity that has been observed across a wide range of cortical regions (Buzsáki and Wang, 2012). The growing body of evidence that long-range GABAergic and glutamatergic/excitatory neurons, across different cortical areas, innervate the same target region (Tomioka et al., 2005, 2015; Tomioka and Rockland, 2007; Lee et al., 2014; Rock et al., 2016; Melzer et al., 2017; Zurita et al., 2018a; Bertero et al., 2019) invite to speculate that these two opposing forces can dynamically contribute to oscillations between different brain areas.

In the cortex, neuronal activity synchronization, especially in the gamma-frequency (30–100 Hz), has been supposed to enable communication between pyramidal neurons across areas of the brain to facilitate learning, attention, and cognitive behavior. Particularly, the circuits underlying gamma oscillations are thought to depend entirely on Parv neurons with local axonal arborization (Buzsáki and Wang, 2012). Buzsáki et al. (2004), using a computational model of excitatory and inhibitory neurons, demonstrated that by adding long-range GABAergic neurons to the neuronal model increased the synchronization of the entire network as was showed by the emergence of a clear oscillation. Despite theoretical and computer modeling studies showing that long-range GABAergic neurons are crucial for cortical oscillations, there is no study yet that has resolute the function of these neurons in cortical oscillation across multiple brain areas. The intrinsic oscillatory properties of CS-Parv neurons mirror the characteristics of striatal fast-spiking neurons, as described by Bracci et al. (2003). Their study revealed that such voltage-dependent oscillations were generated by an intrinsic membrane mechanism that did not require fast synaptic transmission and depended on sodium but not calcium conductance. They also showed how a small additional injected current during the rising phase of oscillation was able to trigger a new burst of an action potential that largely outlasted the current pulse, suggesting that the correct timing of synaptic input on near-threshold membrane potential oscillation can result in long-lasting effects. This result invites to speculate that sodium conductance can be the underlying mechanism of CS-Parv intrinsic generation of gamma-oscillations in membrane potential, and the triggering of the fast-spiking burst. However, an additional study from the cortex (Golomb et al., 2007) and striatum (Sciamanna and Wilson, 2011) showed that not only sodium but also potassium conductance (Kv1 Channels) contributes to both gamma resonance and stuttering properties of the parvalbumin-expressing neurons.

Further experiments aiming at understanding the ionic mechanism and circuit involved in the triggering and termination of CS-Parv action potential bursts, and the channel composition of their subcellular compartments, will be fundamental better to understand their role in long-range GABAergic oscillatory mechanisms. Also, it has been demonstrated striatal local field potentials (LFPs) are characterized by gamma oscillations that are differentially modulated during behavior (e.g., DeCoteau et al., 2007; Berke, 2009). Particularly, Cowan and Wilson (1994) and Stern et al. (1997) have established that some of these striatal LFPs are generated from cortico-striatal excitatory inputs that are also locked in the gamma frequency. Here, we have shown that the striatum receives not only glutamatergic excitatory inputs from the auditory cortex but also inhibitory inputs from parvalbumin-expressing neurons (**CS-Parv inhibitory projections → auditory striatum**). Further investigations focusing on the role of striatal partners of auditory CS-Parv neurons will be fundamental. Can these long-range projections target the direct and/or indirect pathway? Can they exert their functions through direct inhibition on spiny projection neurons or cause disinhibition by targeting local interstriatal GABAergic neurons? How can CS-Parv neurons modulate local and long-range circuits during auditory processing?

Although beyond the target of the present study, it is intriguing to speculate that layer 5 CS-Parv neurons could play a role, through gamma oscillation, in the synchronization between the auditory cortex and striatum. Mainly, layer 5 CC-Parv neurons will provide temporal windows in which active neural assemblies can interact coherently and effectively, therefore acting as high pass filter of concerted network activity not only locally but also in their long-range target(s).

We are proposing that future experiments will provide further insight into the role of the CS-Parv neurons in timing and ratio of excitation and inhibition, two opposing forces in the mammalian cerebral cortex, that can dynamically affect the cortico-striatal dynamic. The new studies will provide a general mechanism of cortico-striatal oscillation involved in reward-learning, and action-selection behavior driven by the auditory stimuli.

## Data Availability Statement

The raw data supporting the conclusions of this article will be made available by the authors, without undue reservation.

## Ethics Statement

The animal study was reviewed and approved by LARC, University of Texas at San Antonio.

## Author Contributions

All authors designed the experiments, performed the experiments, analyzed the data, and wrote the manuscript.

## Conflict of Interest

The authors declare that the research was conducted in the absence of any commercial or financial relationships that could be construed as a potential conflict of interest.

## Footnotes

^1^https://www.janelia.org/open-science/ephus

^2^http://mouse.brain-map.org/static/atlas
